# Immunomodulatory Activities of Body Wall Fatty Acids Extracted from *Halocynthia aurantium* on RAW264.7 Cells

**DOI:** 10.4014/jmb.2007.07032

**Published:** 2020-09-28

**Authors:** Chaiwat Monmai, A-Yeong Jang, Ji-Eun Kim, Sang-Min Lee, SangGuan You, SeokBeom Kang, Tae Ho Lee, Woo Jung Park

**Affiliations:** 1 East Coast Life Sciences Institute, Gangneung-Wonju National University, Gangneung 25457, Republic of Korea; 2 Department of Wellness-Bio Industry, Gangneung-Wonju National University, Gangneung 5457, Republic of Korea; 3 Department of Marine Biotechnology, Gangneung-Wonju National University, Gangneung 25457, Republic of Korea; 4 Department of Marine Food Science and Technology, Gangneung-Wonju National University, Gangneung 2557, Republic of Korea; 5 Citrus Research Station, National Institute of Horticultural and Herbal Science, RDA, Seogwipo 63607, Republic of Korea; 6 Department of Power Plant, Korea Polytechnic College (Mokpo Campus), Muan-gun, Jeollanam-do 58542, Republic of Korea

**Keywords:** *Halocynthia aurantium*, body wall, fatty acids, immune-regulation, NF-κB, MAPK

## Abstract

Tunicates are known to contain biologically active materials and one species in particular, the sea peach (*Halocynthia aurantium*), has not been thoroughly studied. In this study we aimed to analyze the fatty acids profile of the *H. aurantium* body wall and its immunomodulatory effects on RAW264.7 macrophage-like cells. The fatty acids were classified into three categories: saturated fatty acids (SFAs), monounsaturated fatty acids (MUFAs), and polyunsaturated fatty acids (PUFAs). Omega-3 fatty acid content, including EPA and DHA, was higher than omega-6 fatty acids. *H. aurantium* body wall fatty acids exhibited enhanced immune response and anti-inflammatory effects on RAW264.7 macrophage-like cells. Under normal conditions, fatty acids significantly increase nitric oxide (NO) and PGE_2_ production in a dose-dependent manner, thereby improving the immune response. On the other hand, in LPS-treated RAW264.7 cells, fatty acids significantly decreased nitric oxide (NO) and PGE_2_ production in a dose-dependent manner, thereby enhancing anti-inflammatory effects. Fatty acids transcriptionally control the expression of the immune-associated genes, *iNOS, IL-1β, IL-6, COX-2*, and *TNF-α*, via the MAPK and NF-κB signaling cascades in RAW264.7 cells. However, in LPSstimulated RAW264.7 cells, *H. aurantium* body wall fatty acids significantly inhibited expression of inflammatory cytokine; similarly, production of COX-2 and PGE_2_ was inhibited. The results of our present study provide insight into the immune-improving and anti-inflammatory effects of *H. aurantium* body wall fatty acids on macrophages. In addition, our study demonstrates that *H. aurantium* body wall is a potential source of immune regulatory components.

## Introduction

The immune system plays an important role in maintaining homeostasis and preventing disease by destroying harmful foreign substances or organisms and preventing harmful cell changes [1-3]. Macrophages are important immune regulatory cells known to exhibit various biological activities [4] and are associated with cytokine secretion, immune cell recruitment, microbicidal functions, and microbe phagocytosis [5]. Activation of macrophages induces expression of cyclooxygenase-2 (COX-2) and nitric oxide synthase (iNOS), and production of inflammatory cytokines, such as IL-1β and TNF-α, which play crucial roles in inflammation [6], and IL-6, which supports the differentiation of B cells [7]. Macrophage activation pathways are therefore promising targets for disease prevention [8].

Fatty acids such as saturated fatty acids (SFAs), monounsaturated fatty acids (MUFAs), and polyunsaturated fatty acids (PUFAs), have been reported to be beneficial in improving patients with chronic diseases including cancers [9], cardiovascular disease [10], and diabetes [11]. Great attention has been paid to PUFAs, which are classified into two groups according to the first double bond location from the methyl terminus of the fatty acid chain; omega-3 (n-3) and omega-6 (n-6) with double bonds on the third and sixth carbon from the chain terminus, respectively. PUFAs are major structural components of phospholipid membranes and are critical for membrane structure and function [12]. They play important roles in body functions, including the immune system, blood clotting, cholesterol metabolism, and brain development and functioning [13, 14]. In addition, PUFAs modulate inflammatory reactions by producing pro- and anti-inflammatory lipid mediators called ‘eicosanoids’ [15]. An important inflammatory eicosanoid, prostaglandin E2 (PGE_2_), stimulates the pro-inflammatory cytokine IL-6 in macrophage cells, leading to vasodilation and pain. It also provokes the production of diverse cytokines including *TNF-α, IL-1β*, and *IL-6* [16].

As seafood, sea squirts (*Halocynthia roretzi*) are a potential source of various biologically active components such as eicosapentaenoic acid (EPA), docosahexaenoic acid (DHA), carotenoids, taurine, and plasmalogen [17, 18]. According to Murakami, sea squirt carotenoid inhibited immunodeficiency virus reverse transcriptase [19] and repressed both superoxide and nitric oxide generation [20]. Furthermore, acethlene carotenoids contained in sea squirt lipids reduced the secretion of pro-inflammatory cytokines like IL-6 and IL-1β from macrophage-like RAW264.7 cells stimulated by lipopolysaccharides [21]. Another report showed that sea squirt halocynthiaxanthin and fucoxanthinol induced apoptosis in human leukemia and colon cancer cells [22].

Unlike other species of sea squirt, to the best of our knowledge, no research has been done on the benefits of *Halocynthia aurantium* fatty acids. *H. aurantium* is an edible marine invertebrate that inhabits the East Sea, and it is consumed in the countries of East Asia, including Korea and Japan [23]. *H. aurantium* is thought to contain diverse biologically active components [24]. In this study, we explored the immunomodulation effects of *H. aurantium* body wall fatty acids on the macrophage-like RAW264.7 cell line.

## Materials and Methods

### Sea Squirt Samples

**Fig. 1:**
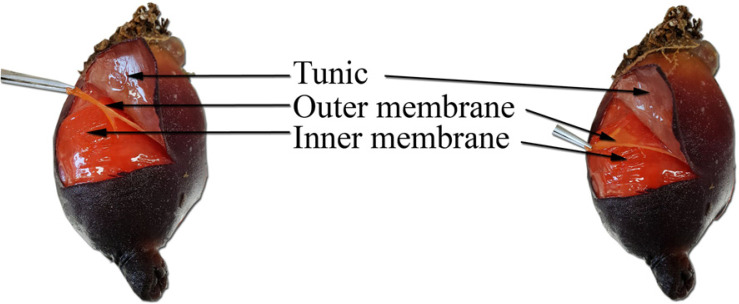
The structure of *H. aurantium*. The body has a tunic (outer protective covering). After removal of the tunic, there is a thin layer which is the outer membrane of the body wall (OM). Inside the OM there is a thicker dermis, the inner membrane of the body wall (IM).

*Halocynthia aurantium* is a valuable benthic marine organism found in the northern region of the East Sea, Korea [25]. The sea squirt prefers attachment to vertical rock faces in subtidal areas at a depth of up to 100 meters [26]. Sea squirt samples were obtained from the East Sea near Gangwon Province, South Korea. After removal of the sea squirt tunic, the outer (Fig. 1) and inner membranes of the body wall were removed for fatty acid extraction.

### Fatty Acid Extraction and Analysis

Fatty acid was extracted from the outer and inner membranes of the body wall (OM and IM) of the sea squirt according to the Garces and Mancha method [27]. The extracted fatty acid was dissolved in DMSO to a final concentration of 20 mg/ml. The experiment was performed in quintuplicate (*n* = 5). Fatty acid methyl ester (FAME) was prepared following the previously described method [28]. Prepared FAMEs were analyzed using gas chromatography (GC) flame-ionization detection (FID) (Perkin Elmer, USA). GC analysis was done using the capillary column (SPTM-2560, 100 m × 0.25 mm i.d., film thickness 0.20 μm). The GC standard is shown in Table 1.

### Macrophage Viability and NO Production Analysis

Macrophage-like RAW264.7 cells were suspended in RPMI-1640 medium (supplemented with 10% FBS and 1% penicillin/streptomycin) and seeded at a concentration of 1 × 10^5^ cells/well. The plate was incubated at 37°C with 5% CO_2_ for 24 h. Different concentrations of OM and IM fatty acids were added to the cultured medium and the plate was incubated for 1 h. Cells were stimulated with and without 1 μg/ml of LPS to activate the inflammatory response and then incubated for another 24 h in the same condition. After incubation, 100 μl of supernatant from each well was analyzed for nitric oxide concentration using Griess reagent (Sigma-Aldrich, USA) [29, 30]. For cultured cells, EZ-Cytox Cell Viability Assay Kit (Daeil Labservice, South Korea) was used to determine macrophage viability [31]. The macrophage proliferation ratio was calculated based on the following formula:

$$\mathrm{Macrophage}\mathrm{viability}\mathrm{ratio}(\%)=\frac{\mathrm{the}\mathrm{absorbance}\mathrm{at}450\mathrm{nm}\mathrm{of}\mathrm{the}\mathrm{test}\mathrm{group}}{\mathrm{the}\mathrm{absorbance}\mathrm{at}450\mathrm{nm}\mathrm{of}\mathrm{the}\mathrm{control}\mathrm{group}}\times 100$$

### RNA Isolation and Reverse Transcription

Total RNA was isolated from the treated RAW264.7 cells (according to the method of macrophage viability and NO production) using TRI Reagent (Molecular Research Center, Inc., USA). RNA was precipitated by adding 100% isopropanol and incubating at 4°C for at least 10 min. The RNA pellet was collected by centrifugation and washing with 75% ethanol. After the last washing, the RNA pellet was dissolved in nuclease-free water and the concentration was measured using a nanophotometer (Implen Inc., Germany). Complementary DNA (cDNA) was synthesized using a high capacity cDNA reverse transcription kit (Applied Biosystems, USA) according to the manufacturer’s instructions.

### Analysis of Immune Gene Expression Using Quantitative Real-Time PCR

The immune-associated genes expression level was evaluated using the QuantStudio 7 Flex Real-Time PCR System (Thermo Fisher Scientific, USA). The qPCR was performed using SYBR Premix Ex Taq II (Takara Bio Inc., Japan) with a 96-well format in a total reaction volume of 20 μl/well. The reaction mixture consisted of 0.4 μM of each specific primer pair (Table 2) and 0.1 ng of cDNA templates. The quantification results were calculated using the 2^-ΔΔCT^ method [32] and compared with β-actin as the reference mRNA.

### Phagocytosis Uptake Assay

RAW264.7 cells were incubated with varying concentrations of *H. aurantium* body wall fatty acids for 24 h. Then, the cells were collected and incubated with 1 mg/ml of FITC-dextran (Sigma-Aldrich, USA) at 37°C for 1 h. The phagocytosis reaction was stopped using ice-cold PBS and then the cells were washed three times with cold PBS. Cellular uptake of FITC-dextran was analyzed using a CytoFLEX Flow Cytometer (Beckman Coulter, Inc., USA).

### Quantification of PGE_2_

The PGE_2_ ELISA Kit (Enzo Life Sciences, USA) was used to determine the production of PGE_2_ in the cell culture medium according to the manufacturer’s instructions. Colorization was done using p-nitrophenyl phosphate substrate, and the optical density was evaluated at 405 nm. The production of PGE_2_ was analyzed based on a standard curve.

### Western Blot Assay

RAW264.7 cells were seeded at the concentration of 2 × 10^6^ cells/well in a 6-well plate and incubated for 24 h. The cells were cultured with varying concentrations of *H. aurantium* body wall fatty acids before stimulating with or without 1 μg/ml of LPS. After 24 h incubation, the cells were collected and the protein was extracted using RIPA buffer (Tech & Innovation, China) containing 0.1% protease inhibitor (Thermo Fisher). The extracted protein was quantified using the Pierce BCA Protein Assay Kit (Thermo Scientific). Thirty micrograms of protein from each sample were collected using SDS-polyacrylamide gel electrophoresis (SDS-PAGE) and transferred to a polyvinylidene fluoride (PVDF) membrane. The western blot assay was carried out as described previously [33]. After membrane blocking, the membrane was incubated with antibodies specific to phospho (p)-NF-κB p65 (1:1,000, Cell Signaling Technology), p-p38 (1:1,000, Cell Signaling Technology), p-ERK1/2 (1:1,000, Cell Signaling Technology), p-JNK (1:1,000, Cell Signaling Technology, USA) and α-tubulin (1:1,000, Abcam, USA). The Pierce ECL Plus Western Blotting Substrate (Thermo Scientific) was used to detect protein signals. The blot was captured using the ChemiDoc XRS+ imaging system (Bio-Rad, USA) and quantified using ImageLab software (version 4.1, Bio-Rad).

### Statistical Analysis

Statistical analysis was performed using ‘Statistix 8.1’ Statistics software. The fatty acid analysis was run in triplicate, and the data were presented as the mean value with standard deviation. Statistical differences were tested using one-way ANOVA and Duncan’s multiple-range test at *p* < 0.05. Macrophage proliferation, NO production, immune-associated gene expression, and production of PGE_2_ results were compared to model control using one-way ANOVA. Differences were considered significant if *p* < 0.01.

## Results

### Fatty Acid Analysis of *H. aurantium* Body Wall

**Fig. 2:**
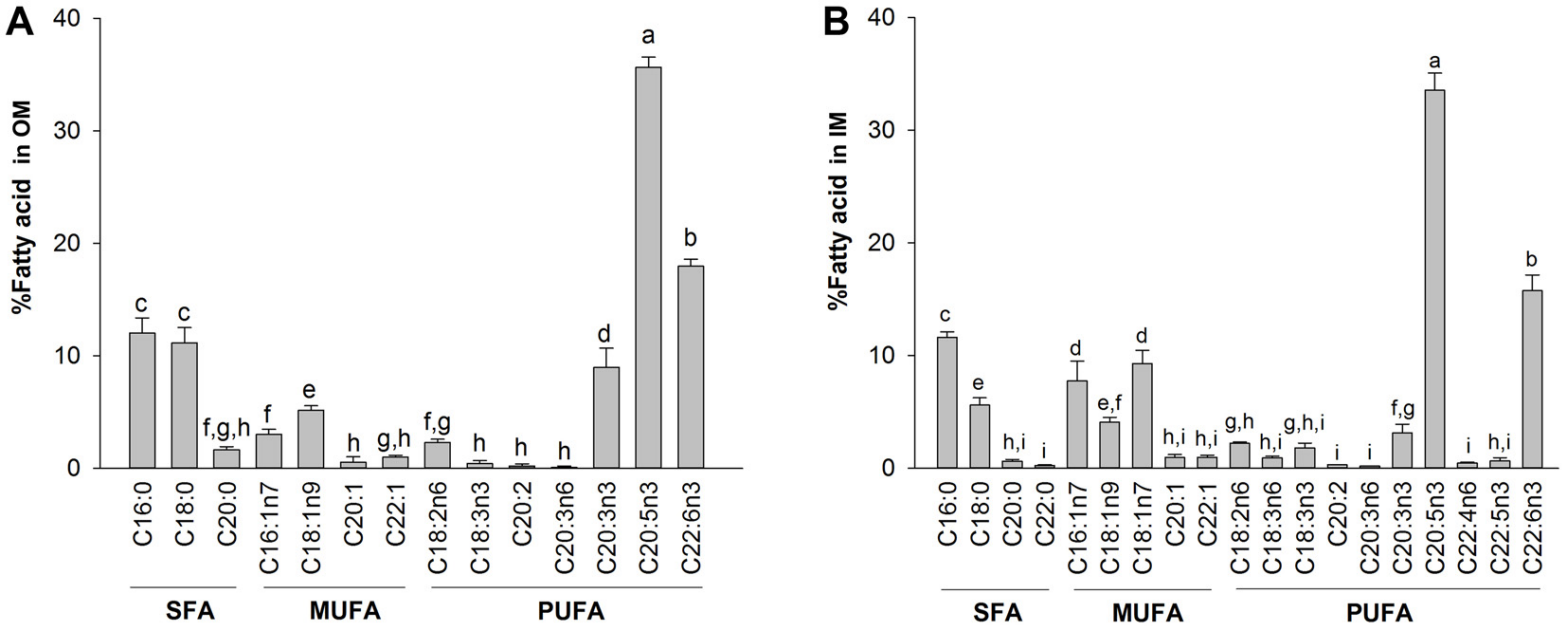
The fatty acid composition of body wall fatty acids extracted from *H. aurantium*, (A) outer membrane (OM), and (B) inner membrane (IM). Data are presented as means±standard deviation. The letters (a–i) indicate significant differences (*p* < 0.05) between the amounts of fatty acids, which were obtained from OM and IM fatty acid.

The extract of total fatty acid from the sea squirt OM and IM amounted to approximately 3.78 ± 0.44% (0.17 ± 0.02 g) and 3.48 ± 0.34% (0.16 ± 0.02 g) of the input raw material, respectively. Total fatty acids from the OM and IM of the sea squirt body walls are shown in Fig. 2. The three groups of fatty acids (SFAs, MUFAs, and PUFAs) from the OM accounted for 24.8 ± 2.5%, 9.7 ± 1.2%, and 65.5 ± 1.6% of the total fatty acids respectively. SFAs, MUFAs, and PUFAs from the IM accounted for 18.0 ± 0.3%, 23.0 ± 2.1%, and 58.9 ± 2.1% of total fatty acids respectively. Both the OM and IM contained higher levels of omega-3 PUFA than omega-6 PUFA. Eicosapentaenoic acid (EPA, 20:5n-3) levels were higher than those of docosahexaenoic acid (DHA, 22:6n-3).

### *H. aurantium* Body Wall Fatty Acid Effect on RAW264.7 Cell Viability

**Fig. 3:**
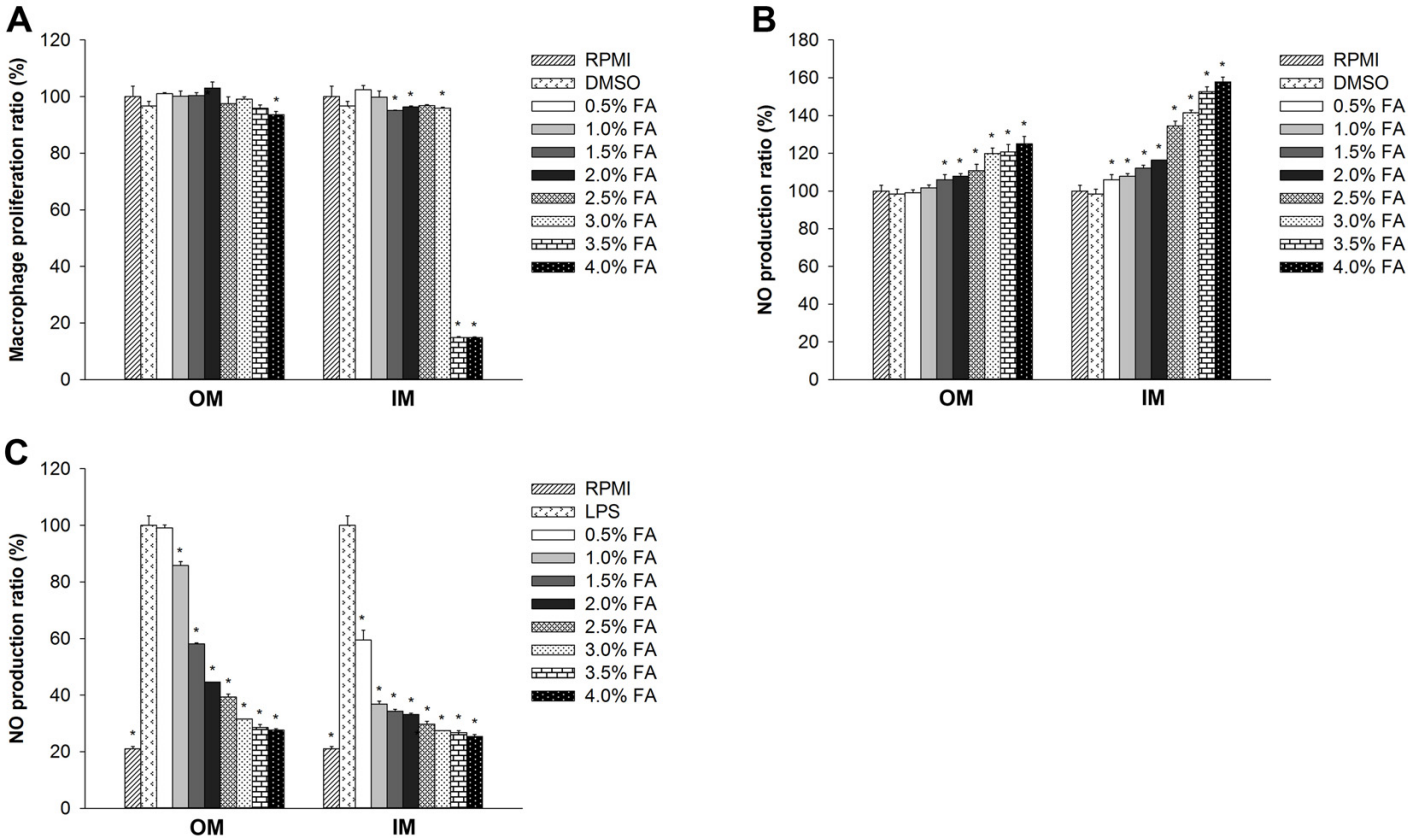
The effect of body wall fatty acids extracted from *H. aurantium*, (A) effect on macrophage viability, (B) effect on NO oxide production in RAW264.7 cells, and (C) effect on NO production in LPS-stimulated RAW264.7 cells. Significant differences at *p* < 0.01 compared to DMSO or LPS.

RAW264.7 cell viability was determined at varying fatty acid concentrations using the EZ-Cytox Cell Viability Assay Kit (Fig. 3A). OM fatty acids did not exhibit cytotoxicity at nearly all experimental concentrations. IM fatty acids showed low cell viability at concentrations between 3.5–4.0%.

### *H. aurantium* Body Wall Fatty Acid Effect on NO Production

To examine the potential immune-enhancing and anti-inflammation effects of *H. aurantium* body wall fatty acids, we evaluated nitric oxide (NO) production of RAW264.7 cells, which is a biomarker for immune regulation [34]. Fig. 3B shows the immune-enhancing effects in which fatty acids increased NO production in a dose-dependent manner. IM fatty acid showed high levels of NO production with high concentrations (2.5–4.0%). On the other hand, Fig. 3C shows the potential anti-inflammatory properties of fatty acids. The inflammatory response was stimulated in RAW264.7 cells, and fatty acids extracted from the body wall significantly repressed the production of LPS-stimulated NO production depending on the concentration.

### *H. aurantium* Body Wall Fatty Acid Effect on Immune-Associated Gene Expression

**Fig. 4:**
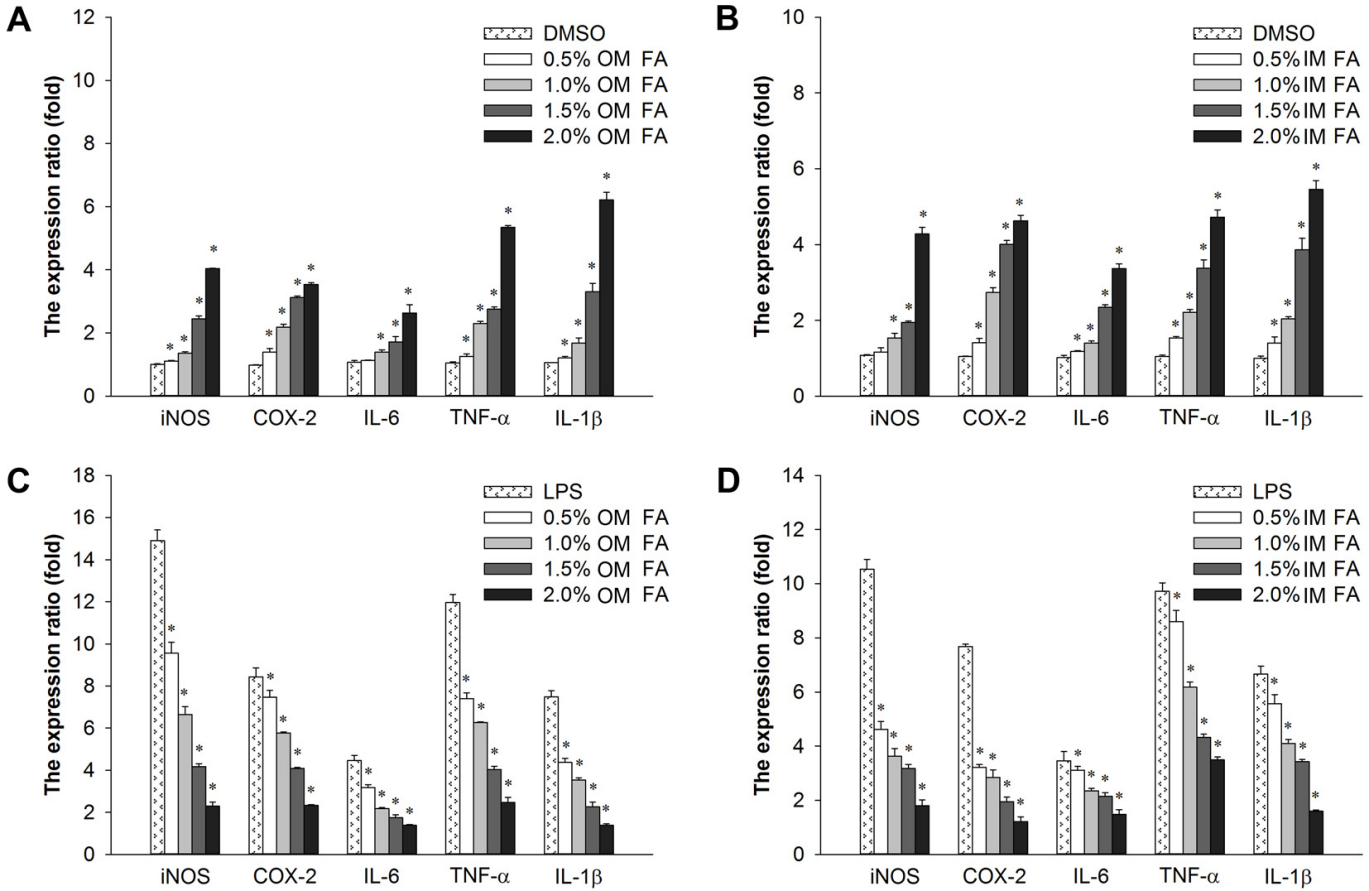
The effect of body wall fatty acids extracted from *H. aurantium* on immune-associated mRNA expression, (A) effect of OM fatty acids on RAW264.7 cells, (B) effect of IM fatty acids on RAW264.7 cells, (C) effect of OM fatty acids in LPS-stimulated RAW264.7 cells and (D) effect of IM fatty acids on LPSstimulated RAW264.7 cells. Significant differences at *p* < 0.01 compared to DMSO or LPS.

Fig. 4 showed the immune-modulation of *H. aurantium* body wall fatty acids via immune-associated gene expression in RAW264.7 cells. In Figs. 4A and 4B, *H. aurantium* body wall fatty acids enhanced the expression of immune-associated genes in RAW264.7 cells in a concentration-dependent manner. The expression of *TNF-α* and *IL-1β* was increased by both sources of body wall fatty acids, while the expression levels of *iNOS, COX-2*, and *IL-6* in IM were higher in IM than in OM. In contrast, Figs. 4C and 4D show that *H. aurantium* body wall fatty acids inhibited the expression level of most immune-associated genes in a dose-dependent manner in LPS-stimulated RAW264.7 cells. The immune-associated expression for most genes was reduced by treatment with *H. aurantium* body wall fatty acids, and *iNOS* and *TNF-α* expression was also reduced with a reduced concentration of body wall fatty acids from both sources.

### *H. aurantium* Body Wall Fatty Acid Effect on Phagocytic Uptake of RAW264.7 Cells

**Fig. 5:**
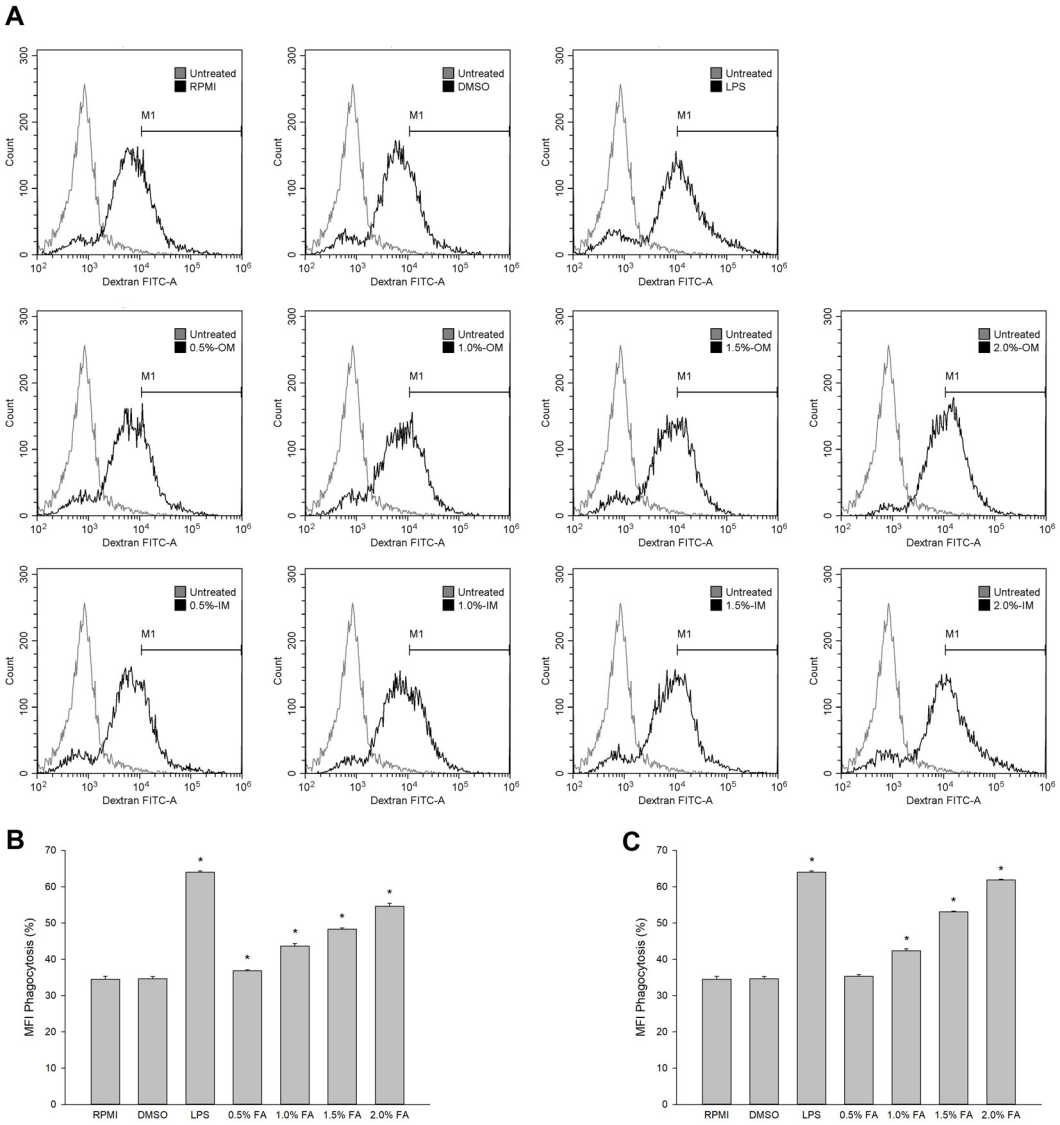
Effect of body wall fatty acids extracted from *H. aurantium* on phagocytic uptake from RAW264.7 cells. (**A**) Representative of flow cytometry analysis of FITC-dextran uptake, (**B**) the percentage of mean fluorescence intensity (MFI) of cells pre-incubated with OM fatty acid, and (**C**) the percentage of mean fluorescence intensity (MFI) of cells preincubated with IM fatty acid. Data are presented as means ± standard deviation (*n* = 3). Significant differences at *p* < 0.01 compared to RPMI.

As shown in Fig. 5, OM and IM fatty acids from *H. aurantium* increased the phagocytosis of the RAW264.7 cells in a dose-dependent manner. Treatment with 0.5% OM significantly increased phagocytosis of cells while treatment of 0.5% IM did not show any changes. However, at high doses of IM, 1.5%, and 2.0%, there were increased numbers of phagocytosis cells than at the same doses of OM.

### *H. aurantium* Body Wall Fatty Acid Effect on PGE_2_ Production

**Fig. 6:**
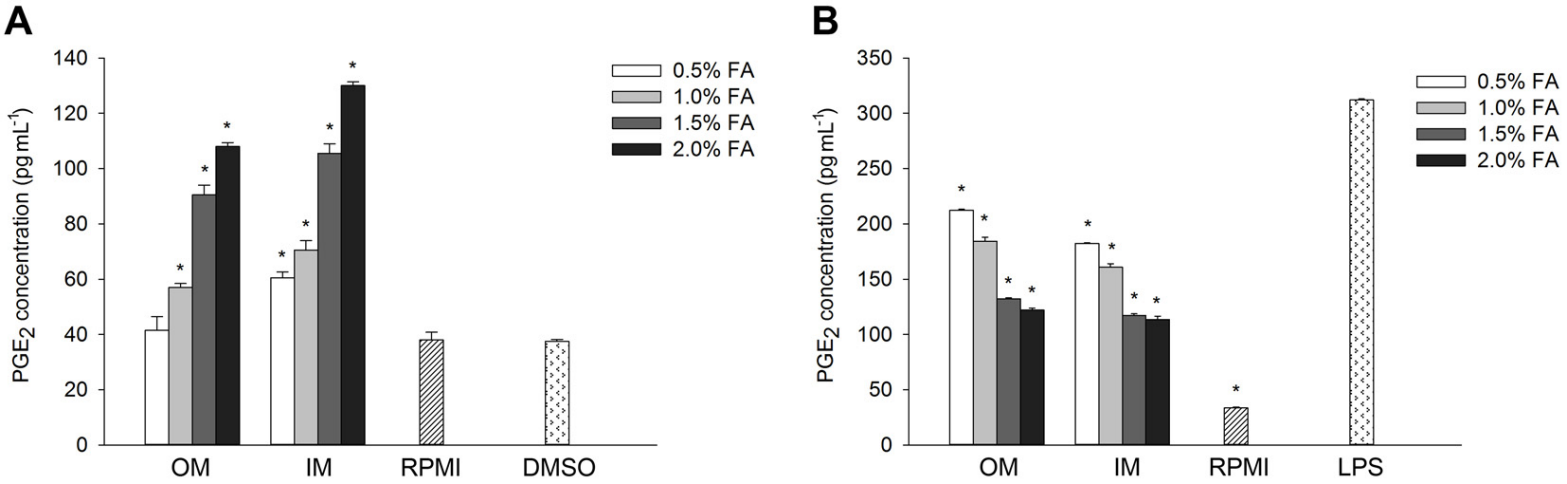
The effect of body wall fatty acids extracted from *H. aurantium* on PGE_2_ production, (A) effect in RAW264.7 cells, and (B) effect in LPS-stimulated RAW264.7 cells. Significant differences at *p* < 0.01 compared to DMSO or LPS.

The immune-enhancing and anti-inflammatory effects of *H. aurantium* body wall fatty acids were analyzed based on the amounts of PGE_2_, an important factor in immune regulation. As shown in Fig. 6A, PGE_2_ production increased with higher concentration of fatty acids in RAW264.7 cells. The level of PGE_2_ production was slightly higher with IM fatty acids than with OM fatty acids, with no significant difference between them. In contrast, fatty acids inhibited the production of PGE_2_ in a dose-dependent manner in LPS-stimulated RAW264.7 cells (Fig. 6B).

### *H. aurantium* Body Wall Fatty Acid Effect on Protein-Associated NF-κB and MAPK Pathways

**Fig. 7:**
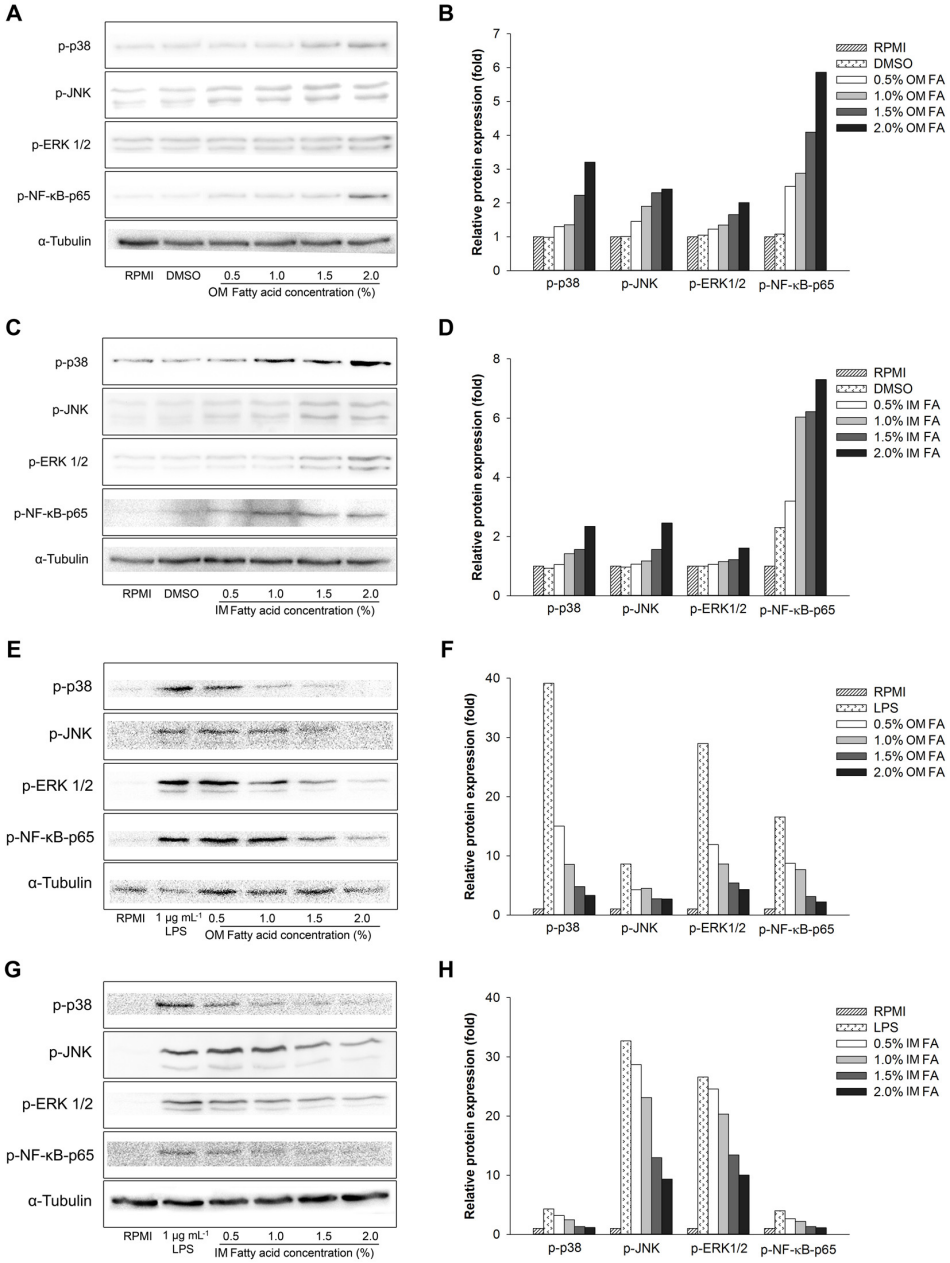
The effect of body wall fatty acids extracted from *H. aurantium* on proteins associated with the NF-κB and MAPK pathways, (A) western blot of OM fatty acids in RAW264.7 cells, (B) relative band intensity of OM fatty acids in RAW264.7 cells, (C) western blot of IM fatty acids in RAW264.7 cells, (D) relative band intensity of IM fatty acid in RAW264.7 cells, (E) western blot of OM fatty acids in LPS-stimulated RAW264.7 cells, (F) relative band intensity of OM fatty acids in LPS-stimulated RAW264.7 cells, (G) western blot of IM fatty acids in LPS-stimulated RAW264.7 cells, and (H) relative band intensity of IM fatty acids in LPSstimulated RAW264.7 cells.

To investigate the cellular immune signaling pathway utilized by *H. aurantium* body wall fatty acids, we evaluated the production of NF-κB and MAPK-associated proteins using western blotting. Figs. 7A–7D showed that *H. aurantium* body wall fatty acids stimulated the phosphorylation of ERK1/2, JNK, and p38 in the MAPK pathway in a dose-dependent manner. Both sources of body wall fatty acids also intensely activated the phosphorylation of NF-κB p-65 in the NF-κB signaling pathway in a dose-dependent manner. The proteins associated with NF-κB and MAPK pathways were also investigated for their effects on anti-inflammatory signaling pathways of LPS-stimulated and *H. aurantium* body wall fatty acid- treated RAW264.7 cells. As shown in Figs. 7E–7H, both body wall fatty acids repressed the phosphorylation of NF-κB p-65, ERK1/2, JNK, and p38 in a dose-dependent manner. Interestingly, OM fatty acids exhibited a higher inhibitory effect on the phosphorylation of p38, ERK1/2, JNK, and NF-κB p-65 in the NF-κB pathway than IM fatty acids; however, there was increased phosphorylation of JNK, and ERK1/2 of the MAPK pathway with IM fatty acids treatment.

## Discussion

Sea squirts are known to contain biologically active components such as carotenoids, taurine, eicosapentaenoic acid (EPA), docosahexaenoic acid (DHA), and plasmalogen [23]. Based on our fatty acid analysis of *H. aurantium* body walls, omega-3 PUFAs, EPA and DHA are the most abundant fatty acids, while SFAs and MUFAs such as PA, SA, and OA are the least abundant. Evidence shows that long-chain polyunsaturated fatty acids such as EPA and DHA exert their anti-inflammatory effects by interrupting TLR signaling and producing anti-inflammatory eicosanoid, stimulated by their incorporation into the plasma membrane [35, 36]. Moreover, EPA and DHA are known to be associated with alteration of cell membrane phospholipid fatty acid composition, disruption of lipid rafts, and deactivation of NF-κB leading to changes in gene expression related to the immune system and fatty acid metabolism [16]. Therefore, *H. aurantium* body walls may be important reservoirs of EPA and DHA, precursors for inflammatory regulation components like eicosanoids and docosanoids [37].

Macrophages are important players in inflammatory response. During acute and chronic inflammation, macrophages trigger the production of NO stimulating the killing of microorganisms within their phagolysosomes [38]. We analyzed macrophage-like cells to understand the immune regulatory effects of *H. aurantium* body wall fatty acids. As shown in Fig. 3A, they did not exhibit cytotoxicity. However, high concentrations (3.5−4.0%) of IM fatty acids led to low RAW264.7 cell viability, indicating that OM fatty acids are safe at concentrations below 4.0%unlike high concentrations of IM fatty acids. Further, we evaluated nitric oxide (NO) and PGE_2_, as important immune functional biomarkers responsible for pain, fever, swelling, and tenderness. Our results showed that the *H. aurantium* body wall fatty acids increased the production of NO and PGE_2_ in a dose-dependent manner. On the other hand, in LPS-stimulated RAW264.7 cells, production of NO and PGE_2_ decreased in a dose-dependent manner. This is an indication that *H. aurantium* body wall fatty acids increased immune regulatory biomarkers NO and PGE_2_ for immune improvement and reduced those biomarkers for anti-inflammatory effects.

Macrophage phagocytosis plays a key role in clearing foreign substances such as pathogenic bacteria [39]. In this study, we measured the phagocytic activity of OM and IM fatty acids by staining dextran with FITC. Fig. 5 shows that OM and IM fatty acids enhance the phagocytic function of RAW264.7 cells in a dose-dependent manner.

NO and PGE_2_ production have the same effect on the expression of immune-associated genes *iNOS, IL-1β, IL-6, TNF-α*, and *COX-2*. These genes are known to tune immune related functions of macrophages, while their gene products, such as inflammatory moderators and associated cytokines, are associated with NF-κB signaling responses through the phosphorylation and degradation of IκBα [40, 41]. Additionally, immune components such as cytokines and stress regulate MAPKs (ERK1/2, JNK, and p38), which are known to regulate cell growth and differentiation [31]. Importantly, MAPK signaling is thought to abate NF-κB signaling, stressing the expression of pro-inflammatory cytokines and inflammatory processes [42, 43].

In our results, fatty acids increased immune-associated genes expression in a dose-dependent manner in non-LPS-treated RAW264.7 cells (Figs. 4A and 4B) and diminished gene expression in LPS-stimulated RAW264.7 cells (Figs. 4C and 4D). The gene expression stimulated NF-κB p-65 and MAPK-associated ERK1/2, JNK, and p38 to control immune reactions for both immune improvement (Figs. 7A−7D) and anti-inflammatory effects (Figs. 7E−7H). This suggests that *H. aurantium* body wall fatty acids modulate immune responses via MAPK and the NF-κB immune-signaling cascade [44-46].

## Conclusions

Our study demonstrated that fatty acids extracted from the outer and inner membranes of sea squirt enhanced the immune response and anti-inflammatory effects of RAW264.7 cells. We evaluated varying responses of key immune biomarkers; NO and PGE_2_, immune-associated gene expression, and MAPK and NF-κB signaling cascade in controlling immune reactions. The results of this study provide insight into the immune regulatory functions of *H. aurantium* body wall fatty acids composed of SFAs, MUFAs, and PUFAs on macrophages. In addition, our study demonstrated that the *H. aurantium* body wall is a potential source of immune regulatory components, and therefore provides a framework for further research on *H. aurantium* fatty acids as a solution for immune modulation.

## Figures and Tables

**Table 1:** Fatty acid retention times in the GC standard.

Peak No.	Retention time (min)	Fatty acid
1	10.397	C4:0
2	11.082	C6:0
3	12.281	C8:0
4	14.251	C10:0
5	15.608	C11:0
6	17.058	C12:0
7	18.753	C13:0
8	20.430	C14:0
9	21.869	C14:1
10	23.605	C16:0
11	26.747	C17:1
12	27.349	C18:0
13	27.934	C18:1n9(t)
14	28.199	C18:1n9(c)
15	28.946	C19:0
16	29.604	C18:2n6(c)
17	30.528	C20:0
18	30.622	C18:3n6
19	31.237	C20:1
20	31.306	C18:3n3
21	32.093	C21:0
22	32.646	C20:2
23	33.645	C20:3n6
24	34.262	C22:1n9
25	34.395	C20:3n3
26	34.470	C20:4n6
27	35.211	C23:0
28	35.753	C22:2
29	36.341	C24:0
30	36.845	EPA
31	37.688	C24:1
32	41.116	DHA

**Table 2:** Sequences of oligonucleotide primers used for macrophage immune-associated genes test.

Gene	Accession No.	Sequence
iNOS	BC062378.1	Forward primer: TTCCAGAATCCCTGGACAAG
		Reverse primer: TGGTCAAACTCTTGGGGTTC
IL-1β	NM_008361.4	Forward primer: GGGCCTCAAAGGAAAGAATC
		Reverse primer: TACCAGTTGGGGAACTCTGC
IL-6	NM_031168.2	Forward primer: AGTTGCCTTCTTGGGACTGA
		Reverse primer: CAGAATTGCCATTGCACAAC
COX-2	NM_011198.4	Forward primer: AGAAGGAAATGGCTGCAGAA
		Reverse primer: GCTCGGCTTCCAGTATTGAG
TNF-α	D84199.2	Forward primer: ATGAGCACAGAAAGCATGATC
		Reverse primer: TACAGGCTTGTCACTCGAATT
β-actin	NM_007393.5	Forward primer: CCACAGCTGAGAGGAAATC
		Reverse primer: AAGGAAGGCTGGAAAAGAGC
